# Insight into norcantharidin, a small-molecule synthetic compound with potential multi-target anticancer activities

**DOI:** 10.1186/s13020-020-00338-6

**Published:** 2020-05-29

**Authors:** Mu-Su Pan, Jin Cao, Yue-Zu Fan

**Affiliations:** Department of Surgery, Tongji Hospital, Tongji University School of Medicine, Tongji University, 389 Xincun Road, Shanghai, 200065 People’s Republic of China

**Keywords:** NCTD, Antitumor agent, Anticancer activities, Mechanism

## Abstract

Norcantharidin (NCTD) is a demethylated derivative of cantharidin, which is an anticancer active ingredient of traditional Chinese medicine, and is currently used clinically as a routine anti-cancer drug in China. Clarifying the anticancer effect and molecular mechanism of NCTD is critical for its clinical application. Here, we summarized the physiological, chemical, pharmacokinetic characteristics and clinical applications of NCTD. Besides, we mainly focus on its potential multi-target anticancer activities and underlying mechanisms, and discuss the problems existing in clinical application and scientific research of NCTD, so as to provide a potential anticancer therapeutic agent for human malignant tumors.

## Background

Since Tu Youyou was awarded the 2015 Nobel Prize in physiology or medicine for the discovery of artemisinin used for malaria treatment, traditional Chinese medicines (TCMs) and natural medicine are getting more attention. A growing body of evidences indicate that TCMs contain anticancer ingredient. Norcantharidin (NCTD), a demethylated derivative of cantharidin which is an active ingredient of TCM—*Mylabris* [1–3], is currently used clinically as an optional anticancer drug in China, because of its relatively synthesized facility, potential anticancer activity, and less side-effects such as myelosuppression, gastrointestinal and urinary tract toxicity [1–5]. Increasing evidences show that NCTD not only effectively inhibited the proliferation of many tumor cells in vitro and in vivo, including hepatoma HepG2 [6–8], SMMC-7721 [8, 9] and BEL-7402 [10, 11], gallbladder cancer GBC-SD cells [12, 13], colon cancer CT26 and HT29 cells [14, 15], breast cancer cells [16, 17], leukemia K562 [18] and HL-60 cells [4, 5, 19], melanoma A375 cells [20], and oral cancer KB cells [21], but also decreased tumor growth and prolonged survival in animal models in vivo [17, 22]. As an efficacious anticancer drug, it has been used to treat hepatic cancer, gastric cancer and leucopenia patients in China for many years. To deepen the understanding of the characteristics and clinical application of NCTD is of great significance for NCTD to work as an anticancer drug in clinic. Here, we review the physiological, chemical, pharmacokinetic characteristics and clinical uses, especially, potential multi-target anticancer activities such as inducing apoptosis, inhibiting proliferation, blocking invasion/metastasis, antiangiogenesis, anti-vasculogenic mimicry, anti-lymphangiogenesis and underlying mechanisms of NCTD, so as to provide a potential anticancer therapeutic agent for human malignant tumors.

## Physiological, chemical and pharmacokinetic characteristics

**Fig. 1:**
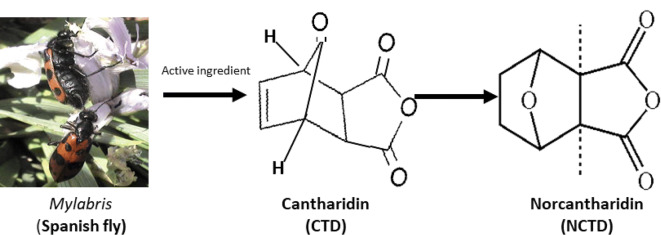
The origin, evolvement and molecular formula of norcantharidin (NCTD). *Mylabris*, also known as Spanish fly, is a traditional Chinese medicine. Cantharidin (CTD), a 7-oxabicyclo [2.2.1] heptane-2, 3-dicarboxylic acid derivative, a natural toxin and the active ingredient with antitumor properties extracted from a traditional Chinese medicine *Mylabris*. NCTD (7-oxabicyclo [2.2.1] heptane-2, 3-dicarboxylic anhydride), with a molecular formula of C_8_H_8_O_4_ and formula weight of 168.15 g/mol, is the demethylated analog and the low-cytotoxic derivative of CTD with antitumor properties

**Fig. 2:**
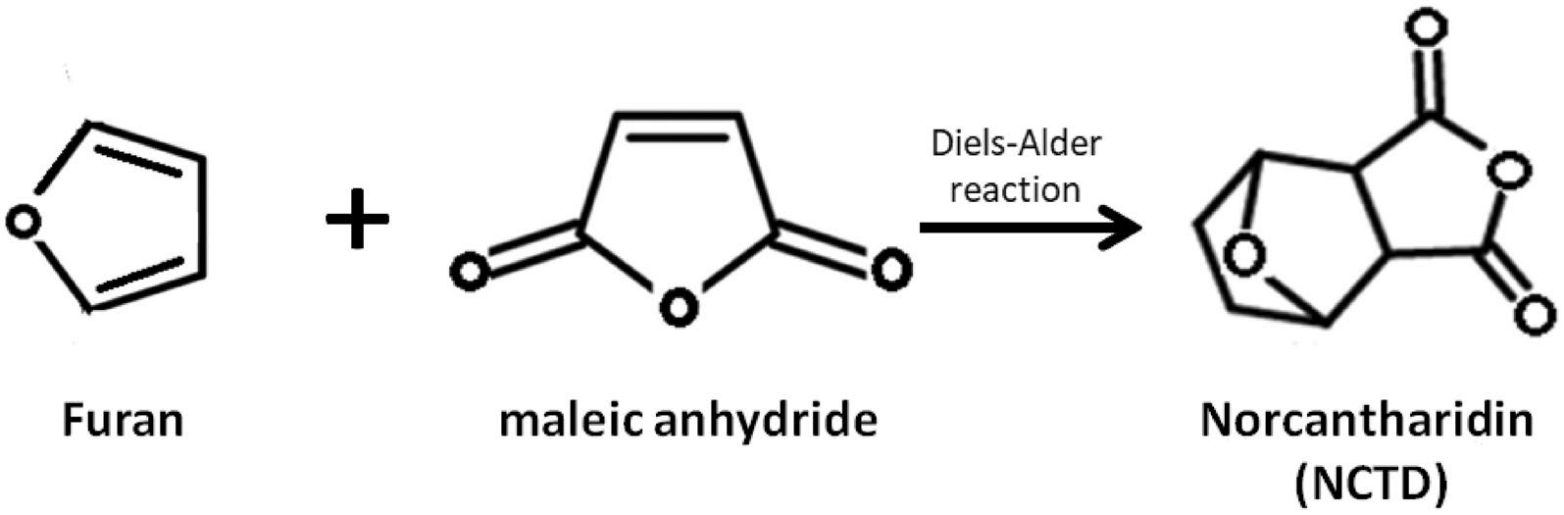
Synthesis of NCTD by furan and maleic anhydride through Diels–Alder reaction. NCTD can be synthesized by furan and maleic anhydride through Diels–Alder reaction under appropriate conditions

Norcantharidin (NCTD, 7-oxabicyclo[2.2.1] heptane-2,3-dicarboxylic anhydride) is a demethylated analogue of cantharidin (CTD). The molecular formula is C_8_H_8_O_4_ and the molecular formula is 168.15 g/mol. NCTD can not only be extracted from TCM *Mylabris* (Spanish fly) [1–4] (Fig. 1), but also can be synthesized from furan and maleic anhydride via the Diels–Alder reaction [23] (Fig. 2). It is a colorless, odorless, slightly irritating crystalline powder, being slightly soluble in water and ethanol, and soluble in hot water and acetone. This small-molecule synthetic compound has low-cytotoxic features and few side effects such as less marrow suppression (myelosuppression), low toxicity of gastrointestinal and urinary tract, because of removing 1,2 methyl groups on the chemical structure of CTD [1–5].

In pharmacokinetics, radionuclide and whole-body autoradiography showed that NCTD was rapidly absorbed by intragastric administration in mice with ^3^H-norcantharidin, reached a higher concentration within 15 min and 2 h after dosing in the kidney, liver, tumor, stomach, intestines, heart and lung. NCTD was highly distributed in the bile duct, liver, kidney, heart and lung by intravenous administration, reached the peak concentration in liver and cancer tissues within 15 min after dosing. After 6 h, the concentration decreased significantly by being excreted from the urethra. Most of drugs were excreted from the kidneys within 24 h, and were rarely accumulated in the various organs of the body [24]. Thus, NCTD is less likely to cause drug accumulation poisoning.

## Clinical uses

As an efficacious anticancer drug, NCTD has been used to treat cancer patients clinically in China for many years. Two thousand years ago, *Mylabris* (Spanish fly), a traditional Chinese medicine, was used to treat “abdominal mass” in China [1–4]. Later, an active ingredient of *Mylabris*—CTD was artificially extracted and be used to treat many human tumors as a natural toxin [1–4]. Afterwards, in order to alleviate side effects of CTD such as gastrointestinal and urinary tract toxicity, NCTD was extracted from CTD, or was synthesized from furan and maleic anhydride [1–4, 23]. Now, NCTD is clinically used as a routine anticancer drug in China.

Clinical indications of NCTD include: (1) It is used to treat patients with digestive tumors, such as hepatocellular cancer, esophageal cancer, gastric cancer, and colorectal cancer and it shows better curative effect; (2) It is used to treat other cancer patients, such as lung, breast and ovarian cancers and has certain curative effect; (3) Also, it is used as premedication or in combination with other antineoplastic drugs. In addition, NCTD can also be used for hepatitis, liver cirrhosis and leukopenia.

Usage of NCTD includes oral, intravenous administration and local injection. For oral, 5–15 mg (most dose can be added to 30 mg) NCTD is used for one time, 3 times a day, 1 months for 1 courses, generally 3 courses. For intravenous infusion or intravenous drip, 10–20 mg a day, added to the 5% glucose injection 250–500 ml, in a slow drop by intravenous drip; or added to the 5% glucose injection 10–20 ml, by slow intravenous injection; 1 month for 1 treatment course. And for local injection, 20–40 mg/times, once a week, 2–4 times for 1 courses.

Growing clinical evidences demonstrated that NCTD was an efficacious anticancer drug for cancer patients. Table 1 illustrates the clinical uses of NCTD and the related results [25–48]. No matter NCTD is used alone via oral, intravenous administration, intro-tumor injection, or in combination with chemotherapy, radiotherapy and other therapies such as interventional therapy (IVT), transcatheter arterial chemoembolization (TACE) and TCMs can reduce tumors, improve symptoms and life quality, alleviate side effects, and prolong survival time in most patients with mid-advanced stage tumors such as hepatocellular cancer, esophageal cancer, gastric cancer, lung cancer, ovarian cancer, non-Hodgkin lymphoma and so on [25–48]. Thus, NCTD is believed as a useful adjunct anticancer drug in clinical treatment of mid-advanced stage tumors and in the prevention of post-operational recurrent tumors.

**Table 1:** Clinical uses of NCTD in treatment of cancer patients and the related results and outcomes

Cancers	n	Therapies and usages of NCTD				Efficient (CR + PR)	Symptoms or LQ improving	Tumor marker decreasing	Tumor size reducing	Survival time prolonging	Side effects alleviating	References
		Method	Dose	Course	Group							
PHC (I–III stage)	244	po or iv	10 mg, tid; or 5–20 mg, qd, iv	1–18 month			58.6%	(AFP) 39%	Yes, 40.7%	MST 7 month 1 year SR 30%	Yes, WBC↑72%	[25]
PHC (I–III stage)	86	iti	iti, 20 mg, qw po, 10 mg, tid	4 weeks 3–6 months	iti vs. po		Yes, *P *< 0.05	Yes, *P *< 0.05	Yes, *P *< 0.05	1 year SR, *P *< 0.05	Yes, *P *< 0.05	[26]
PHC (I–III stage)	41	iv + po	10 mg, qd, iv 5 mg, qd, po	1 month 1–3 months			Yes, *P *< 0.05		Yes, 31.7%	MST 6.8 month 1 year SR 17.7%	Yes, WBC↑59%	[27]
PHC (II–III stage)	76	po + Chem	po, 10 mg, tid Chem., FAM regimen	3–12weeks	po + Chem vs. Chem		–	(AFP) 39%	66% vs. 35% *P *< 0.05	NS	NS	[28]
PHC (II–III stage)	75	po + TCM	10 mg, tid, po GFL, 10 tab, tid, po	3 months	NCTD + GFL vs. NCTD or GFL		Yes	CR + PR, *P *< 0.05 84% vs. 7% or 53%	Yes	1 year SR, *P *< 0.05 41% vs. 27% or 12%	Yes	[29]
PHC (Ad)	54	iti	NCTD-P407, 2–4 ml, qw	2–3 weeks	NCTD-P407 *vs*. TACE		Yes, *P *< 0.05	NS	NS	NS	Yes, *P *< 0.05	[30]
PHC (Ad)	56	iti	NCTD-P407, 2–4 ml, qw Ethanol 4-8 ml, qw	2–3 weeks 6–8 weeks			NS		NS	1 year SR, *P *< 0.05	NS	[31]
PHC (Ad)	80	po + IVT	IVT, 1/m × 4 po, 5–10 mg, tid	4 months 3 months	po + IVT vs. placebo + IVT placebo + IVT			Yes, *P *< 0.05	Yes, *P *< 0.05		Yes, *P *< 0.05	[32]
PHC (Ad)	43	iv + Chem	30 mg, iv, qd × 10 5-FU + CF regimen	20 days	iv + Chem vs. Chem		Yes, *P *< 0.05		Yes, *P *< 0.05		Yes, *P *< 0.05	[33]
PHC (Ad)	47	iv + TACE	10–20 mg, iv qd	1–2 months	iv + TACE vs. TACE		Yes, *P *< 0.05		Yes, *P *< 0.05			[34]
PHC (Ad)	60	po + TCM	10–15 mg, po, tid	3 months	po + TCM vs. TCM	Yes, *P *< 0.05	Yes, *P *< 0.05					[35]
PHC (Ad)	79	po + TCM	15 mg, po, tid	2 month	po + TCM vs. Chem/IVT		Yes, *P *< 0.05		Yes, *P *< 0.05	MST, 16 month vs. 11 month *P *< 0.01	Yes, *P *< 0.05	[36]
SHC	60	po + Chem	15 mg, po, tid	3 months	po + Chem vs. Chem		Yes, *P *< 0.05		Yes, *P *< 0.05		Yes, *P *< 0.05	[37]
GC (Ad)	50	iv + Chem	30 mg, iv qd × 7–10	6 weeks	iv + Chem vs. Chem	NS	Yes, *P *= 0.02		NS		Yes, *P *< 0.05	[38]
GC II-III (post-op.)	82	po + Chem	15 mg, po, tid PLF regimen	6 months 4 weeks × 6	po + Chem vs. Chem					3 year SR, *P *< 0.05 3 year RR, *P *< 0.05	Yes, *P *< 0.05	[39]
EC	58	iv + RT	30 mg, iv, qd × 10 RT,200GY, qd × 5	4 weeks 2 weeks	iv + RT vs. RT	Yes, *P *< 0.05	Yes, *P *< 0.05		Yes, *P *< 0.05		Yes, *P *< 0.05	[40]
CC (III stage)	264	iv + RT	20–30 mg, iv, qd RT,20GY, qd × 5	6–8 weeks	iv + RT vs. RT				NS	Yes, *P *< 0.05	Yes, *P *< 0.05	[41]
NHL	86	iv + Chem	15–25 mg, iv, qd CHOP regimen	2 weeks	iv + Chem vs. Chem	NS	Yes, *P *< 0.05		NS	NS	Yes, *P *< 0.05	[42]
NHL	57	iv + Chem	30–40 mg, iv, qd CTOP regimen	2 weeks	iv + Chem vs. Chem	NS	Yes, *P *< 0.05		NS		Yes, *P *< 0.05	[43]
LC (Ad)	60	iv + Chem	20 mg, iv, qd × 7 CVI regimen	9 weeks	iv + Chem vs. Chem				Yes, *P *< 0.05		NS	[44]
NSCLC (Ad)	50	iv + Chem	20 mg, iv, qd × 7 DP regimen		iv + Chem vs. Chem				Yes, *P *< 0.05		Yes, *P *< 0.05	[45]
NSCLC (III-IVstage)	85	iv + Chem	60–100 ml, iv, qd × 14 PTC protocol	8 weeks	iv + Chem vs. Chem	Yes, *P *< 0.05	Yes, *P *< 0.05		Yes, *P *< 0.05		Yes, *P *< 0.01	[46]
NSCLC (III-IVstage)	180	iv + Chem	30 mg, iv, qd × 21 GC protocol	9 weeks	iv + Chem vs. Chem	Yes, *P *= 0.007			Yes, *P *< 0.05		Yes, *P *< 0.05	[47]
NSCLC (Ad)	80	iv + Chem	40 ml, iv, qd × 14 DDP protocol	8 weeks	iv + Chem vs. Chem	Yes, *P *< 0.05	Yes, *P *< 0.01		Yes, *P *< 0.05		Yes, *P *< 0.01	[48]

NCTD, norcantharidin; PHC, primary hepatic cancer; SHC, secondary hepatic cancer; GC, gastric cancer; EC, esophageal cancer; CC, cervical cancer; NHL, non-Hodgkin lymphoma; LC, lung cancer; NSCLC, non-small cell lung cancer; Ad, advanced; Chem., chemotherapy; RT, radiotherapy; IVT, interventional therapy; TCM, traditional Chinese medicine; P407, Poloxamer 407; po, per os; iv, intravenous drip; iti, intro-tumor injection; TACE, transcatheter arterial chemoembolization; qd, one a day, quaque die; tid, three times a day, ter in die; qw, one a week; LQ, life quality, Karuafsky score; MST, median survival time; SR, survival rate; CR, complete response; PR, partial response; *P *< 0.05, statistically significant difference; NS, no significant difference

## Multi-target anticancer activities and underlying mechanisms

The multi-target anticancer activities and underlying mechanisms of NCTD in treatment of different cancer models and cell lines have been reported. Here, we systematically review the potential anticancer activities and underlying molecular mechanisms of NCTD in vitro and in vivo.

### Inhibiting proliferation and inducing apoptosis

In recent years, a large number of researches have been carried out to study the effects of NCTD on inhibiting proliferation and inducing apoptosis in different cancer models (Table 2). NCTD has a cytotoxic effect on a variety of tumor cells. Significant anti-proliferative and apoptotic effects are observed in NCTD-treated tumor cells [7, 49, 50]. At the same time, relevant studies have confirmed that NCTD has no myelosuppression and can induce hematopoiesis via bone marrow stimulation while exerting its anticancer activity [4, 5]. NCTD has no effect on the viability of normal peripheral blood mononuclear cells (MNC) [51, 52]. These are incomparable advantages over many traditional anticancer drugs. In addition, NCTD has a synergistic effect with a variety of anticancer drugs, such as cisplatin and gefitinib [53, 54].

**Table 2:** Relevant researches of NCTD on inhibiting proliferation and inducing apoptosis

Cancers	Cell lines	Basic mechanisms	Pathways	Accompanying roles	Experiment	References
Leukemia	K562	DNA synthesis inhibition; G2/M phase cell-cycle arrest			In vitro	[18]
	HL-60	G2/M cell-cycle arrest and apoptosis	Inducing apoptosis via a caspases- dependent pathway, regulated by JNK activation signaling			[19]
	Jurkat	S phase cell-cycle arrest; activation of cytochrome *c*, caspase-9, -3; PARP cleavage	Regulation of ATM	With no effect on the viability of normal MNCs		[51]
	Jurkat T	G2/M phase cell-cycle arrest, down-regulating the expression of calcineurin, reducing calcineurin phosphatase activity	Activation of P38 and ERK1/2	With no myelosuppression		[52]
	HL-60	S and G2/M-phase arrest;DNA synthesis inhibition				[55]
	Jurkat, Ramos	Inducing the degradation of Cdc6				[65]
	Jurkat	Decreasing β-catenin protei	Inhibiting Wnt/β-catenin signaling			[70]
	HL-60	Inhibiting DNA replication, and induce apoptosis and caspase-3-dependent cleavage of Cdc6				[133]
	MV4-11	Modulating the expression of several molecules, including HLF, SLUG, NFIL3 and c-myc		With no myelosuppression, inducing haemopoiesis	In vivo In vitro	[4]
	K562, HL-60	DNA synthesis inhibition; G2/M phase cell-cycle arrest; producing interleukin (IL)-1β, colony stimulating activity (CSA) and tumor necrosis factor (TNF)-alpha	Inhibition of PP2A	Transient leukocytosis, less nephrotoxic and phlogogenic side-effects; stimulating hematopoiesis		[5]
	L1210	Inhibiting the serine/threonine protein PP2A		Without myelosuppression, inducing haemopoiesis		[62]
	Z138, Mino	G2/M, G1 cell-cycle arrest, upregulating caspase-3, -8, and -9, suppressing NF-κB-regulated gene products, such as cyclin D1, BAX, survivin, Bcl-2, XIAP, and cIAP	Inhibiting PI3K–Akt–NF-κB signaling pathway			[72]
Hepatocellular cancer	HepG2	Xenograft growth inhibition		Prolonging host survival	In vivo	[50]
	HepG2		Activation of ERK and JNK; modulation of NF-kappa B and AP-1		In vitro	[6]
	HepG2 Hep3B Huh-7	M-phase cell-cycle arrest; phosphorylation of p21, Cdc25C; regulation of cyclin B1-associated kinase activity; phosphorylation of Bcl-2 and Bcl-X(L), activation of caspase-3, -9				[7]
	SMMC-7721 BEL-7402	Inducing the activation of caspase-9, -3 and the cleavage of PARP, and downregulating the expression of Bcl-2, Bcl-X(L) and Mcl-1.				[11]
	HepG2	Cytotoxic effect				[49]
	Hep3B	Downregulating TGF-β1 and Smad7, up-regulated Smad4	Altering TGF-β1/Smads signaling	With cisplatin synergistic effect		[53]
	HepG2	G2/M phase cell-cycle arrest, upregulating Bax, and downregulating Bcl-2		With EVO synergistic effect		[56]
	BEL-7402	M phase cell-cycle arrest; decreasing Bcl-2 expression				[58]
	HepG2	Inducing the degradation of Cdc6				[65]
	HepG2	Inhibiting pre-RCs assembly, inducing degradation of Cdc6 and Mcm2, inhibiting the nuclear translocation of Mcm6, G1/S phase cell-cycle arrest, inhibiting DNA replication	Inhibiting pre-RCs assembly via degrading initiation protein Cdc6, Mcm2, and Mcm6	With Cdc6 depletion synergistic effect		[66]
	SMMC-7721	Upregulating caspase-3, cytochrome *c*, AIF, and Bax, downregulating Bcl-2	Activation of JNK and mitochondrial pathways			[134, 135]
	HepG2	Downregulating Bcl-2, upregulating Bax, reduction of Bcl-2/Bax ratio	Caspase-3, and -9 activities			[136]
	HepG2	An increase in ROS production, loss of mitochondrial membrane potential and release of cytochrome *c* (cyto-*c*) from the mitochondria to the cytosol and downregulating Bcl-2, upregulating Bax levels. Increasing caspase-9, -3 and PARP	Through ROS generation and mitochondrial pathway			[3]
	Hep3B with deficiency of p53.	G(2)M or G(0)G(1) phase cell-cycle arrest, activation of caspase-3, -10	Activation of a p53-independent pathway (caspase-3 and -10) via TRAIL/DR5 signal transduction			[137]
	HepG2	Downregulating LC3-II, an autophagosome marker; upregulating Bax, cytochrome *c*, caspase-3, -9, PARP, ROS production; disrupting MMP	Inhibiting autophagy via ROS generation and mitochondrial apoptosis pathway activation	Atg5 siRNA enhances the anticancer action		[138]
	HepG2 SMMC-7721	Inhibiting of Mcl-1, thus enhancing the release of cytochrome *C*, ABT-737, inducing apoptosis		Solving the ABT-737 drug resistance problem		[139]
	SMCC-7721 SK-Hep-1	G2/M phase cell-cycle arrest; upregulating FAM46C, mitigating DEN-initiated HCC in mice; inhibiting Ras, p-MEK1/2, p-ERK1/2	Up-regulating FAM46C and inhibiting ERK1/2 signaling		In vivo In vitro	[57]
	Hep3B		Inhibiting PP5 via activating AMPK signaling			[140]
	HepG2 HepG2/ADM hepatoma Hepal-1	Inhibiting cell viability, decreasing CD4+ CD25+ T cells, downregulating FoxP3 in vitro; suppressing tumor formation, downregulating Tregs, FoxP3, CTLA-4, TGF-β, IL-10 in vivo	Downregulating regulatory T cells accumulation	With CLSO synergistic effect		[141]
Gallbladder cancer	GBC-SD	Inhibiting PCNA and Ki-67 expression			In vitro	[12, 67, 142]
	GBC-SD	Inhibiting PCNA, Ki-67, cyclin D1, Bcl-2, Survivin; upregulation of p27, Bax			In vivo In vitro	[143, 144]
	GBC-SD	Inhibiting cyclin D1, Bcl-2, Survivin; upregulating p27, Bax; S phase cell- cycle arrest				[145]
Colorectal cancer	Colo205 HT-29 SW480	G2/M phase cell-cycle arrest, activation of CD95 receptor/ligand and caspase 8			In vitro	[59]
	CT26	Cell cycle arrest in the S and G2/M phases, inducing anoikis-mediated apoptosis	JNK activation			[60]
	Six cell lines		Caspase-3, -8, -9 and MAPK activity			[68]
	HT-29		Inhibiting integrin αvβ6-ERK			[146]
	HCT116, HT29	G2/M phase cell-cycle arrest; downregulating EGFR, p-EGFR, c-Met, p-c-Met, and cyclinD1, Rb, CDK-4; increasing cleaved PARP and caspase-3	Affecting cell cycle- and apoptosis-related signaling	Substituting for gefitinib		[147]
Breast cancer	MCF-7	Repressing cell adhesion to platelets via downregulating α2 integrin	Activating protein kinase C pathway via PP2A inhibition	Inhibiting adhesion and migration	In vitro	[63]
	MCF-7		Inhibiting MAPK and the dephosphorylation of erk1, 2			[148]
	ER-HS-578T ER + MCF-7		Activation of MAPK and STAT pathways			[149]
	Bcap-37	Increased ROS, decreased MMP, induced DNA damage and reduced G1, G2/M peak				[150]
	MDA-MB-231 MDA-MB-468 BT-549 SKBR-3 MCF-7 BT474		Dual inhibition of pAkt and pERK1/2 signaling		In vitro In vivo	[16]
	Highly-metastatic MDA-MB-231	G2/M phase cell-cycle arrest; up-regulating Bax, down-regulating Bcl-2, Bcl-2/Bax ratio, p-Akt, NF-kappaB	Inhibiting the Akt and NF-kappaB signaling	Suppressing tumor growth in vivo		[73]
Gastric cancer	AGS	G0/G1 phase cell-cycle arrest; increasing ROS production, cytochrome *c*, AIF and Endo G release; upregulating BAX, BID, caspase-3, -8, -9; downregulating MMP, caspase-4, -12	Through mitochondria- and caspase-dependent pathways		In vitro	[151]
Melanoma	A375-S2	Caspase-3, -9 activation and Bax upregulaton and Bcl-2 downregulation			In vitro	[152]
	A375-S2		Activation of JNK and p38 MAPK			[153]
	U266	Potentializing the chemosensitivity to ADR	Regulating NF-κB/IκBα signaling pathway and NF-κB-regulated gene products including survivin, Bcl-2, Bax and VEGF	With ADR synergistic effect		[154]
	WM115A, 1205Lu Sbcl2, WM35	Increased cytochome c, Bax and caspase-3, decreased Bcl-2 and NF-κB2	Activation of a TR3 dependent pathway	Improving survival	In vitro In vivo	[20]
		Downregulating IKKα and p-IκBα, inducing the accumulation of IκBα and inhibiting activation of NF-κB, potentializing the chemosensitivity to BTZ	Inhibiting NF-κB signaling pathway	With BTZ synergistic effect		[155]
NSCLC	EGFR mutation − A549 EGFR mutation + PC9	G2/M phase cell- cycle arrest, enhancing the anticancer effects of gefitinib and cisplatin		With gefitinib and cisplatin synergistic effect	In vitro	[54]
	A549 H1299 Calu6	Repressing YAP and its downstream targets CYR61 and CTGF; arresting cell cycle, inducing senescence	Repressing YAP signal pathway	Inhibiting EMT, motile, invasion via enhancing E-cadherin and decreasing fibronectin/vimentin		[80]
	A549	Downregulating Bcl-2, upregulating Bax, reducing Bcl-2/Bax ratio and viability		With trichostatin A, celecoxib, lovastatin, synergistic effect	In vitro In vivo	[157]
Oral cancer	KB cell	Induced significant cytotoxicity			In vitro	[21]
	SAS, Ca9-22	Activation of caspase-9, enhancing Bax, downregulating Bcl-2, Bcl-XL				[108]
Medulloblastoma	DAOY, UW228	Loss of β-catenin activation; reduce of β-catenin expression	Inhibition of Wnt/β-catenin signaling	Ability to cross the blood–brain barrier	In vitro In vivo	[71]
Glioma	U87, C6	Inhibiting phospho-MEK, phospho-ERK, Bcl-2 and Mcl-1	Blocking Raf/MEK/ERK pathway		In vitro	[157]
Neuroblastoma	SH-SY5Y		Inhibiting MAPK and the dephosphorylation of erk1,2		In vitro	[148]
	SK-N-SH	Uppressing proliferation and cloning ability G2/M phase cell-cycle arrest; inducing mitophagy, autophagy; reducing MMP; downregulating cyclin B1, Cdc2, TOM20, SQSTM1/p62, p-AKT, mTOR; upregulating p21, beclin1, LC3-II, caspase-3, -9, p-AMPK; regulating Bax/Bcl-2, Bax/Mcl-1	The AMPK, AKT/mTOR, and JNK/c-Jun signaling pathways are widely involved in these processes via activation of JNK/c-Jun pathway			[158]
Cervical cancer	HeLa	Inducing the degradation of Cdc6.			In vitro	[65]
	HeLa	Up-regulation of caspase-3, -8, -9, and Bax; down-regulation of Bcl-xL.	Activation of ERK and JNK.			[159]
	HeLa	G2/M cell-cycle arrest; downregulating ΔΨ(m), Bcl-2, cyclin B and cdc2; upregulating Bax, cytochrome *c*, p21 and p-cdc25c	Activating p38-NF-κB signaling pathway; p38-NF-κB-promoted mitochondria- associated apoptosis and G2/M cell cycle arrest			[160]
Bladder cancer	TSGH 8301	S, G1phase cell-cycle arrest; upregulating caspase-3, -8, -9 and Fas, FasL, Bax, Bid, cytochrome *c*, and ROS production; downregulating ΔΨ(m), ERK, JNK, p38	Activation of ROS-modulated Fas receptor, caspse-3, -8, -9 mitochondrial -dependent and -independent pathways		In vitro	[161]
Prostate cancer	DU145	Inhibiting DNA replication and pre-RCs, inducing mitotic catastrophe	Blocking ATR-dependent checkpoint pathway; degrading initiation protein Cdc6	With paclitaxel synergistic effec	In vitro	[162]
	DU145	Downregulating PCNA, MnSOD; destructing MMP, ROS-mediated DNA damage; depleting ATP; activating AMPK	ROS-mediated mitochondrial dysfunction and energy depletion			[163]
		Increasing autophagy; inducing autophagic cell death, cell proliferation arrest; upregulating Beclin-1; suppressing miR-129-5p	Inducing autophagy-related cell death through Beclin-1, upregulation by miR-129-5p suppression			[164]
	22Rv1, Du145	Increased oligonucleosomal formation, PARP cleavage; upregulating cytochrome *c*, caspase-3, -8, -9, Fas, DR5, RIP, TRADD; increased ratios of pro-/anti-apoptotic proteins and decreased expression of IAP family member proteins, including cIAP1 and survivin	Inducing both intrinsic and extrinsic apoptotic pathways			[165]
		Mitochondria dysfunction, modulating Akt signaling via increasing nuclear translocation and interaction with Mcl-1	Suppressing Mcl-1 via epigenetic upregulation of miR-320d		In vitro In vivo	[166]
Osteosarcoma	143B, SJSA	Inducing G2/M cell cycle arrest	Blocking the Akt/mTOR signaling pathway		In vitro	[167]
	MG63 HOS	The induction of autophagy, the triggering of ER stress and the inactivation of the c-Met/Akt/mTOR pathway	The inhibition of the c-Met/Akt/mTOR signaling pathway		In vitro In vivo	[22]
Glioblastoma	RT-2 U251	G(2)/M phase arrest and post-G(2)/M apoptosis in RT-2 cell line		Adenoviral p53 gene therapy enhances chemosensitivity of tumor cells to NCTD.	In vitro	[168]
Giant cell tumor of bone (GCTB)		Suppressing the PI3K/AKT signaling pathway through upregulating the expression of miR-30a	Modulating the miR-30a/MTDH/AKT cell signaling pathway		In vitro	[169]

**Fig. 3:**
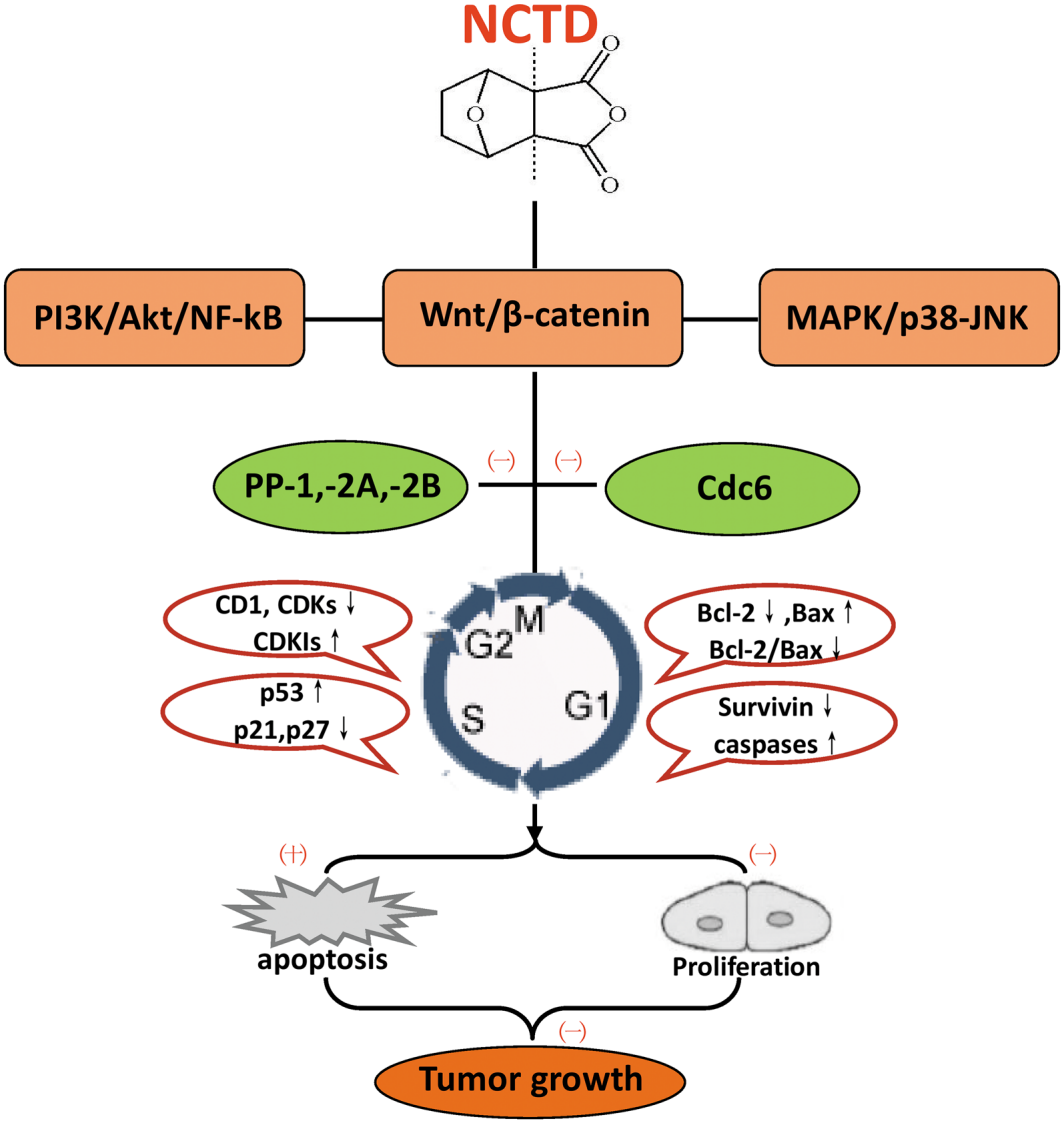
The “multi-points priming” mechanisms of NCTD on inhibiting proliferation and inducing apoptosis. NCTD: norcantharidin; PI3K: phosphoinositide 3 kinase; NF-κB: nuclear factor-kappa B; MAPK: mitogen-activated protein kinase; JNK: Jun N-terminal kinase; PP1: protein phosphatase type 1; PP2A: protein phosphatase 2A; PP2B: protein phosphatase 2B; Cdc6: cell division cycle protein 6; CD1: cyclin D1; CDKs: cyclin-dependent kinases; CDKIs: cyclin-dependent kinase inhibitors; Bcl-2: B-cell lymphoma-2; (−): Inhibition; (+): Promotion or inducing

The anti-proliferation and pro-apoptotic effects of NCTD depend on the complex interactions between different molecules (Fig. 3). On the one hand, the inhibitory effect of NCTD on proliferation is mainly achieved through cell cycle arrest and inhibition of DNA synthesis by inhibiting the expression of cyclins, cyclin-dependent kinases (CDKs) and increasing the expression of cyclin-dependent kinase inhibitors (CDKIs, such as p21^Cip/Waf1^, p27^kip1^); On the other hand, NCTD can also induce apoptosis by increasing the expression of pro-apoptotic protein such as P53, Bax, Caspases, and reducing the expression of anti-apoptotic proteins such as Bcl-2 (B-cell lymphoma-2) and survivin. These mechanisms have been confirmed in a variety of tumor cell lines such as leukemia K562 and HL-60 [18, 55], hepatoma HepG2, SMMC-7721 and BEL-7402 [56–58], colorectal cancer CT26 and HCT-15 cells [59, 60], etc. It is generally believed that serine/threonine protein phosphatases, such as protein phosphatase type 1 (PP1), protein phosphatase-2A (PP2A) and protein phosphatase-2B (PP2B), play important roles in intracellular signal transduction, whose inhibition is an excellent target for the development of novel anti-cancer agents [5, 61, 62]. Some studies have confirmed that NCTD, as a PP2A inhibitor, can inhibit cancer cell proliferation and induce apoptosis by inhibiting the activity of PP2A [5, 62, 63]. In addition, DNA replication-initiation protein Cdc6 (cell division cycle protein 6) is an effective target to disturb DNA replication [64]. Other studies have found that NCTD can inhibit cell proliferation by inducing Cdc6 degradation [65, 66]. In gallbladder cancer, it was reported that NCTD inhibited the expression of GBC-SD cell proliferation-related gene proteins PCNA (proliferating cell nuclear antigen) and Ki-67, this may be one of the mechanisms by which NCTD inhibit the proliferation and growth of tumor cells [12, 67].

NCTD inhibited proliferation and induced apoptosis in cancer cells is dose- and time-dependent [51, 55], and is regulated by both extrinsic and intrinsic signaling pathways [34]. MAPK (mitogen-activated protein kinase) can be divided into four subfamilies: ERK (extracellular regulated protein kinases), p38, JNK (Jun N-terminal kinase) and ERK5. MAPK-related signaling pathways are widely involved in NCTD-induced apoptosis [68]. For instance, NCTD-induced apoptosis in leukemia HL-60 cells is regulated by activating JNK signaling [19], and apoptosis in hepatocellular cancer HepG2 cells induced by NCTD is dependent on ERK and JNK activity [6]. The Wnt/β-catenin signaling pathway is considered to be another target for antitumor drugs [69]. Some studies have shown that NCTD can reduce the proliferation of leukemia Jurkat cells by inhibiting Wnt/β-catenin signaling [70]. Due to the ability to cross the blood–brain barrier, NCTD can also significantly inhibit the growth of medulloblastoma through Wnt/β-catenin signaling pathway [71]. In addition, NCTD can inhibit the expression of the proliferation-related protein cyclin D1, downregulate the expression of anti-apoptotic protein, and upregulate the expression of pro-apoptotic protein by blocking PI3K (phosphoinositide 3 kinase)/Akt/NF-κB (nuclear factor-kappa B) pathway [72, 73]. So, the PI3K/Akt/NF-κB pathway has been shown to be another signal pathway for the regulation of NCTD-mediated anti-proliferation and pro-apoptosis.

### Inhibiting tumor invasion/metastasis

Two major protein families are involved in NCTD against tumor invasion and metastasis, including matrix metalloproteinases (MMPs) and adhesion molecules [74]. The MMP family, particularly MMP-2 and MMP-9, has gelatinase activity and is capable of proteolytic cleavage of plasminogen in extracellular matrix [75]. Cell adhesion molecules such as α-catenin and b-catenin have the function of adhering tumor cells to other cellular and matrix components [76], both of them play an important role in local invasion and distant metastasis.

**Fig. 4:**
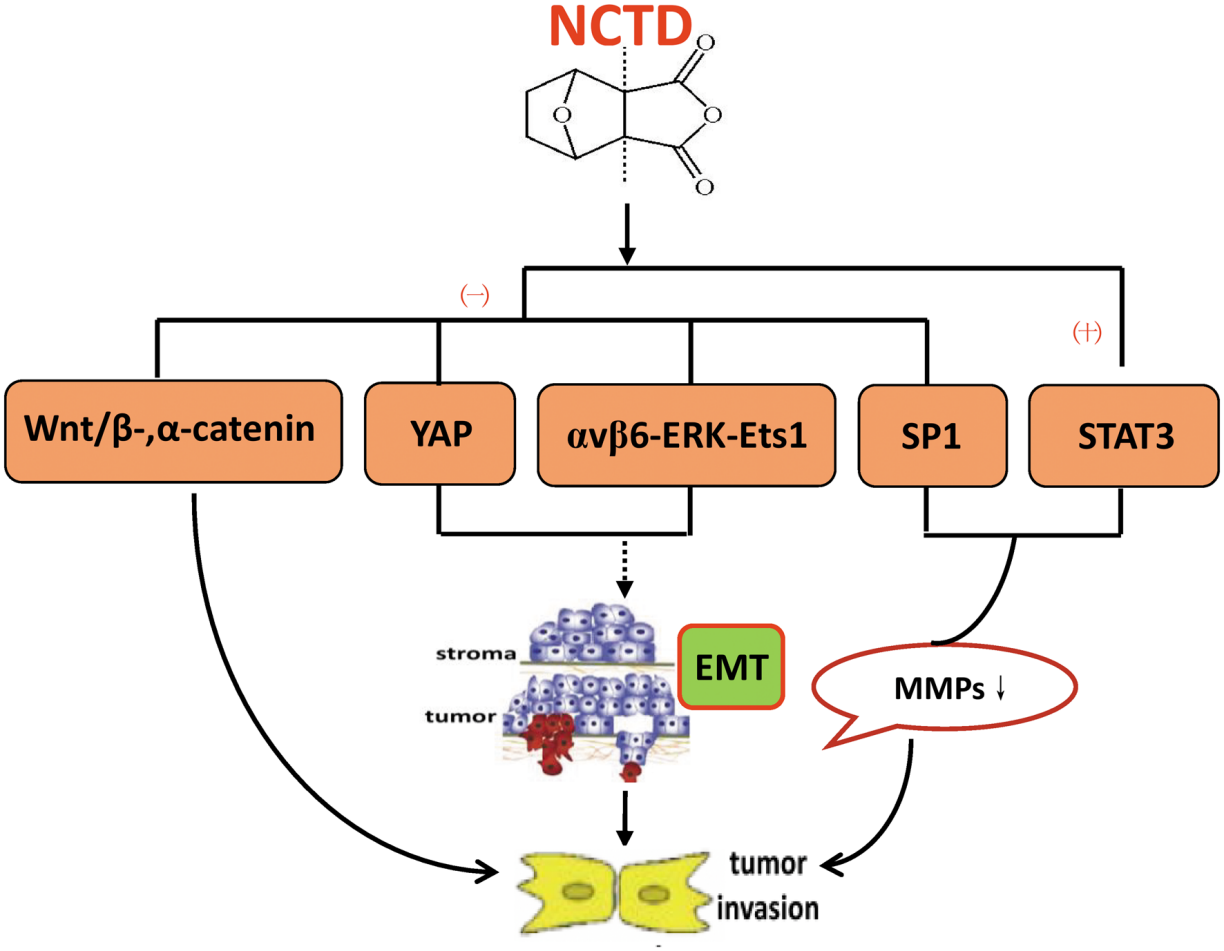
Underlying regulatory targets of NCTD against invasion and metastasis. NCTD: norcantharidin; YAP: Yes-associated protein; ERK: extracellular regulated protein kinases; Ets1: E-Twenty-Six-1; Sp1: specificity protein 1; STAT1: signal transducers and activators of transcription 1; MMPs: matrix metalloproteinases; EMT: epithelial–mesenchymal transition. (−): Inhibition; (+): Promotion or inducing

It has been confirmed that NCTD has anti-invasion and anti-metastasis effects in many kinds of tumor cells (Table 3). Some experiments indicated that NCTD reduces the activity of MMP-2 and MMP-9 by upregulating the transcription factor STAT1 (signal transducers and activators of transcription 1) and inhibiting the transactivation of Sp1 (specificity protein 1), thereby inhibiting the invasion and metastasis of tumor cells [77, 78]. Another study showed that NCTD has the ability to reduce the expression of α-catenin and β-catenin in colorectal cancer CT26 cells, suggesting that the anti-invasive and anti-metastatic activity of NCTD may be related to the regulation of these adhesion molecules [75]. Furthermore, epithelial–mesenchymal transition (EMT) is widely involved in the invasion and metastasis of malignant epithelial tumors [79]. NCTD inhibits the EMT process in non-small cell lung cancer, colorectal cancer and hepatocellular cancer cells via the αvβ6-ERK-Ets1 (E-Twenty-Six-1) signaling pathway blocking and NCTD-mediated Yes-associated protein (YAP) inhibition [78, 80, 81]. These regulatory mechanism of NCTD against tumor invasion and metastasis is detailed in Fig. 4.

**Table 3:** Relevant researches of NCTD against invasion and metastasis for multiple cell lines in different cancer models

Cancers	Cell lines	Basic mechanisms	Pathways	Accompanying roles	Experiment	References
Gallbladder cancer	GBC-SD	Upregulating TIMP-2 and MMP-2/TIMP-2 ratio, downregulating MMP-2			In vitro	[142]
Colorectal cancer	CT26	Downregulating MMP-9 and gelatinase; inhibiting the DNA-binding activity of Sp1	Inhibiting Sp1 transcriptional activity		In vitro	[77]
	HT-29 WiDr	Downregulating αvβ6, MMP-3, MMP-9, N-cadherin, vimentin, p-ERK, p-Ets1; up-regulating E-cadherin	Inhibiting EMT by blocking αvβ6-ERK-Ets1 signaling pathway			[78]
	CT26	Down-expressing MMP-2, -9 and Desmoglein, N-cadherin, α- and β-catenin; reducing pulmonary metastasis.		Prolonging mice survival	In vitro In vivo	[74]
NSCLC	A549 PC9	Inhibiting migration; enhancing the anticancer effects of gefitinib and cisplatin	Not altering p-EGFR	With gefitinib and cisplatin synergistic effect	In vitro	[54]
	A549 H1299 Calu6	Interfering the YAP-mediated cell progression and metastasis; inhibiting EMT, motile, invasion via enhancing E-cadherin and decreasing fibronectin/vimentin; repressing YAP and its downstream CYR61, CTGF	Repressing YAP signal pathway			[80]
	A549	Suppressing migration	Inhibiting p-Akt, NF-κB	With trichostatin A, celecoxib, lovastatin, synergistic effect	In vitro Ex vivo	[156]
Breast cancer	MCF-7	Inhibiting adhesion and migration, repressing cell adhesion to platelets via downregulating α2 integrin	Activating protein kinase C pathway via PP2A inhibition. via protein kinase C pathway-dependent, downregulation of α2 integrin		In vitro	[63]
Hepatocellular cancer	Huh7 SK-Hep1	Downregulating MMP-9, u-PA, p-ERK1/2, NF-kB, FAK; upregulating PAI-1 and TIMP-1	Inhibiting the phosphorylation of ERK1/2 and NF-kB signaling pathway		In vitro	[170]
	SMMC-7721, MHCC-97H	Suppressing cell motility and invasiveness; up-regulating FAM46C; suppressing TGF-β/Smad signaling, EMT	Up-regulating FAM46C via brocking EMT process and TGF-β/Smad signaling			[9]
	HCCLM3 SMMC-7721	Inhibiting IL-6-induced EMT and cell invasiveness, and JAK/STAT3/TWIST signaling	Inhibiting IL-6-induced EMT via JAK2/STAT3/TWIST signaling			[81]
Osteosarcoma	MG63 HOS	Inhibiting the expression of MMP-2 and MMP-9			In vitro In vivo	[22]
Giant cell tumor of bone (GCTB)		Inhibiting the EMT process	Modulating the miR-30a/MTDH/AKT cell signaling pathway		In vitro	[169]

### Anti-angiogenesis and anti-vasculogenic mimicry

Angiogenesis and effective blood supply are basic conditions for tumor growth and metastasis [82]. Multiple angiogenic growth factors and cytokines play important roles in regulating tumor angiogenesis, such as vascular endothelial growth factor (VEGF) and its corresponding receptor, thrombospondin (TSP), angiogenin (Ang), and tissue metalloproteinase inhibitor (TIMP) family. In gallbladder and colorectal cancer, it has been confirmed that NCTD can inhibit angiogenesis, induce apoptosis of vascular endothelial cells, downregulate the expression of angiogenic factors such as VEGF, VEGFR-2 (vascular endothelial growth factor receptor-2), Ang-2, and upregulate the expression of anti-angiogenic factors such as TSP and TIMP-2 [83–86]. So, NCTD may be a potential anti-angiogenic drug for cancer treatment.

Tumor vasculogenic mimicry (VM) refers to a novel tumor blood supply pattern that occurs in certain highly aggressive malignancies and is associated with poor clinical outcomes and poor prognosis [87]. TIMP-2 has anti-VM activity in some highly aggressive malignancies [88]. Furthermore, the PI3-K (phosphatidylinositol 3-kinase)/MMPs (matrix metalloproteinases)/Ln-5γ2 (laminin 5γ2) and EphA2 (ephrin type a receptor 2)/FAK (focal adhesion kinase)/Paxillin signaling pathways are two critical pathways for the control of VM formation [89], while MMP-2 and MT1-MMP (membrane type 1-matrix metalloproteinase) are key molecules and important mediators of these two pathways, regulating VM formation in invasive malignant cells [90]. NCTD is believed as a potential anti-VM active drug, its anti-VM mechanisms mainly involves two aspects: NCTD downregulates the expression of MMP-2 and MT1-MMP via inhibiting EphA2/FAK/Paxillin signaling pathway, thereby enhancing the anti-VM activity of TIMP-2; in turn, a decrease in MMP-2 and MT1-MMP activity inhibits PI3-K/MMPs/Ln-5γ2 signaling and exerts an anti-VM effect on malignant cells [13, 91–93].

### Anti-lymphangiogenesis

Lymphatic metastasis is one of the important metastatic pathways of tumors, and tumor lymphatic tube formation (lymphangiogenesis) plays an important role in tumor growth, metastasis and prognosis [94]. Lymphatic endothelial growth factors, including two members of the VEGF family, VEGF-C and VEGF-D, as well as their cognate receptor VEGFR-3, are the main regulators of tumor lymphangiogenesis and is of great significance in tumor lymph node metastasis [95–97]. In recent years, some researchers have reported that NCTD is an effective lymphangiogenesis inhibitor. The basic mechanism of NCTD anti-lymphangiogenesis refers to directly or indirectly downregulate the expression of VEGF-C, VEGF-D and VEGFR-3 at protein and mRNA levels, which has been proved in human lymphatic endothelial cells (HLECs) and human colonic adenocarcinomas (HCACs) [98–100]. In addition, NCTD in combine with sorafenib or mF4-31C1 enhanced the ability of anti-lymphangiogenesis in human colonic adenocarcinomas [100].

**Fig. 5:**
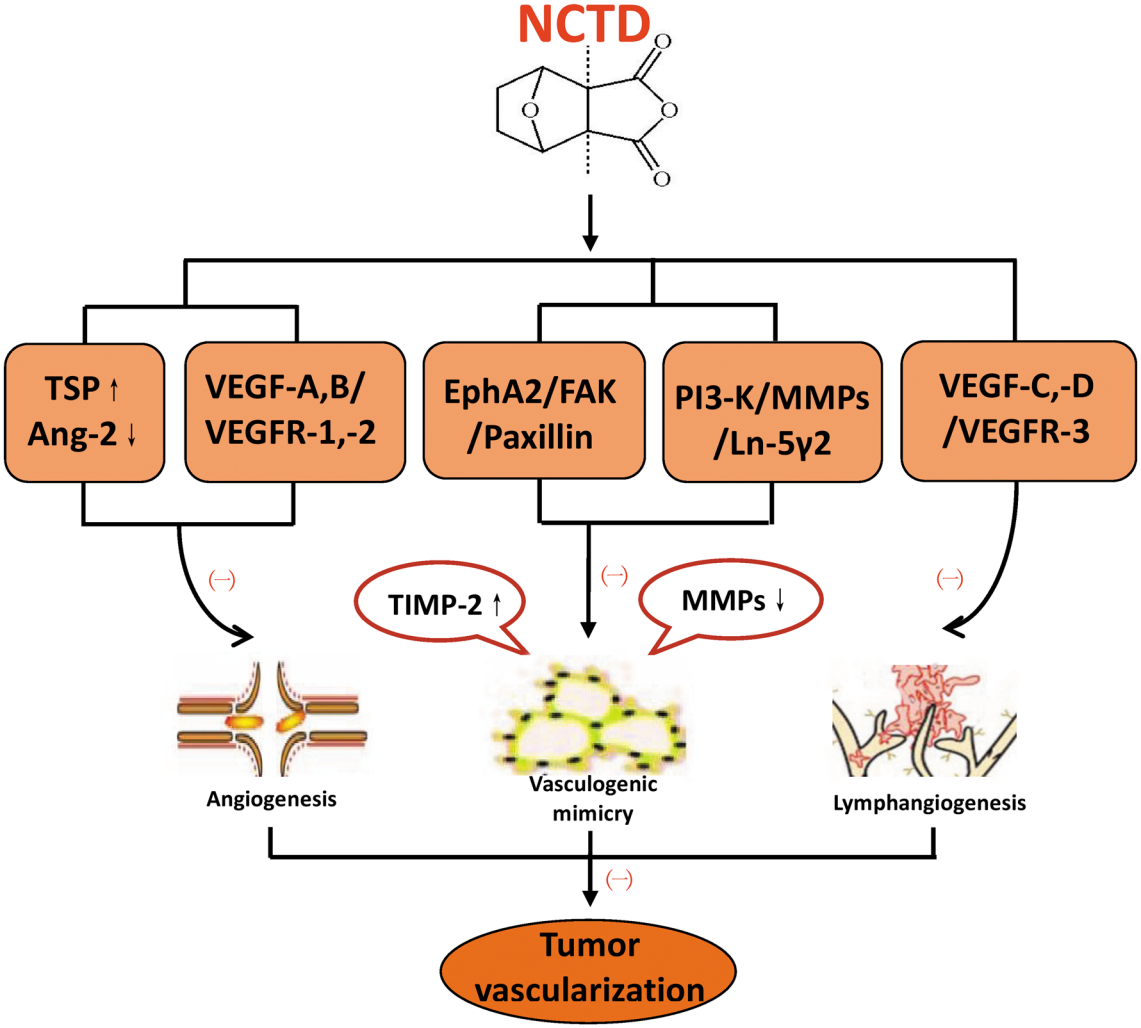
The “more targets” mechanisms of NCTD against tumor vascularization (angiogeneses, VM and lymphangiogenesis). NCTD: norcantharidin; TSP: thrombospondin; Ang-2: angiogenin-2; VEGF: vascular endothelial growth factor; VEGFR: vascular endothelial growth factor receptor; EphA2: ephrin type a receptor 2; FAK: focal adhesion kinase; PI3-K: phosphatidylinositol 3-kinase; MMPs: matrix metalloproteinases; Ln-5γ2: laminin 5γ2; TIMP: tissue metalloproteinase inhibitor. (−): Inhibition; (+): Promotion or inducing

The relevant researches and mechanisms of NCTD inhibiting tumor vascularization (Angiogenesis, VM and lymphangiogenesis) are summarized in Table 4 and Fig. 5.

**Table 4:** Relevant studies of NCTD anti-angiogenesis, anti-VM, and anti-lymphangiogenesis

Anticancer activities	Cancers	Cell lines	Basic mechanisms	Pathways	Accompanying roles	Experiment	References
Anti-angiogenesis	Gallbladder cancer	GBC-SD	Inhibiting capillary-like tube formation of HUVECs in vitro; destroying angiogenesis and CAM capillaries; decreasing xenograft MVD and vascular perfusion in vivo; downregulating VEGF, Ang-2; upregulating TSP, TIMP-2		Prolonging xenograft-mice survival	In vitro	[84]
		GBC-SD	Lower MVD and PCNA/apoptosis ratio, smaller tumor volume; down-regulating VEGF and Ang-2, and up-regulating TSP and TIMP2; MVD positively correlating with VEGF, Ang-2n and negatively correlating with TSP and TIMP2			In vitro In vivo	[83]
	Colorectal cancer	HCT116	Inhibiting xenograft growth and tumor angiogenesis in vivo; reducing migration, adhesion and vascular network tube formation of HUVECs in vitro; downregulating VEGF and VEGFR-2	Downregulating VEGF and VEGFR-2		In vivo	[85]
		CT26	Inhibiting viability, adhesion, migration, capillary-like tube formation of HUVECs, and the release of pro-angiogenic factors from HUVECs; inducing anoikis; down-regulating VEGF, integrin β1, vimentin, p-JNK and p-ERK	Down-regulating VEGF and inhibiting MAPK (JNK/ERK) signaling	Without renal or hepatic toxicity	In vitro In vivo	[14]
		LOVO	Inhibiting VEGF-induced proliferation, migration, invasion, capillary tube formation of HUVECs and LOVO proliferation; inhibiting tumor angiogenesis and tumor growth in vivo; inhibiting VEGFR2/MEK/ERK pathway	Blocking VEGFR2/MEK/ERK			[86]
Anti-VM	Gallbladder cancer	GBC-SD	Inhibiting proliferation, invasion, migration, VM formation in vitro and in vivo; downregulating EphA2, FAK and Paxillin	Blocking the EphA2/FAK/Paxillin signaling pathway	Prolonging xenograft mice survival	In vitro In vivo	[13]
		GBC-SD	Inhibiting proliferation, growth, invasion, migration and VM formation in vitro and in vivo; downregulating MMP-2, MT1-MMP, PI3-K, Ln-5γ2	Suppression of the PI3-K/MMPs/Ln-5γ2 signaling pathway			[91]
		GBC-SD	MMP‑2, MT1‑MMP relating tumor VM In vitro; a poor survival in VM^+^ patients with high MMP‑2, MT1‑MMP expression; inhibiting tumor growth, VM formation, VM hemodynamic in vivo; inhibiting proliferation, invasion, migration and VM‑like networks in vitro; downregulating MMP‑2 and MT1‑MMP in vivo and in vitro; thus, enhancing TIMP‑2 antitumor and anti‑VM activities	Enhancing TIMP-2 anti-VM via downregulating MMP-2 and MT1-MMP	With TIMP-2 synergistic effect; prolonging xenograft mice survival		[92]
	Melanoma	A375		Suppressing MMP-2 expression		In vitro In vivo	[83]
Anti-lymphangiogenesis	HLEC	HDLECs	Inhibiting proliferation, migration, invasion, lymphatic tube formation (lymphangiogenesis), inducing apoptosis; downregulating VEGF-C, VEGF-D and VEGFR-3 expression	Blocking VEGF-C,-D, VEGFR-3		In vitro	[98]
		HDLECs	Inhibiting growth, lymphatic tube formation; inducing apoptosis; downregulating VEGF-C and VEGF-D expression	Downregulating the expression of VEGF-C and VEGF-D			[99]
	Colorectal cancer	HT-29	S-phase cell-cycle arrest; Inhibiting proliferation, migration, invasion, lymphatic tube formation in vitro and tumor growth and lymphangiogenesis in vivo; downregulating Ki-67, Bcl-2, LYVE-1, D2-40, CK20 and their LMVD, and VEGF-A, VEGF-C, VEGF-D, VEGFR-2 and VEGFR-3 in vitro and in vivo	Blocking the VEGF-A,-C,-D, VEGFR-2, -3 “multi-points priming” mechanisms	With mF4-31C1 or Sorafenib synergistic effect	In vitro In vivo	[100]
	AML	TSC-null cell 21-101	Inhibiting proliferation of TSC2−, TSC2+ cells with rapamycin		An additive effect between rapamycin and NCTD in inhibiting lymphangiogenesis	In vitro	[171]

### Overcoming multi-drug resistance

Multi-drug resistance (MDR) refers to tumor cells develop resistance to anti-tumor drugs, as well as producing cross-resistance to other antineoplastics with different structures and mechanisms [101]. As one of the main problems in clinical tumor chemotherapy, MDR directly affects the efficacy of chemotherapy drugs and even lead to treatment failure [102].

In human breast cancer cells, NCTD may overcome MDR through inhibiting sonic hedgehog (Shh) signaling and its downstream MDR-1/P-gp expression [103], which has been shown to increase resistance to a variety of structurally unrelated antitumor drugs [104]. Bcl-2 family proteins Bcl-2 and Bcl-xL are resistant to multiple chemotherapeutic agents in a variety of cell lines [105–107], and it was reported that NCTD downregulated the expression of Bcl-2 and Bcl-xL in oral cancer cells [108]. In addition, Bcl-2 family inhibitors ABT-737 and ABT-263 are two promising anticancer agents with anticancer activity against a variety of cancer cells [109, 110]. NCTD significantly enhances ABT-263 and ABT-737-mediated anticancer activity, and overcomes the increased ABT-737 resistance caused by elevated Mcl-1 levels in cancer cells [111–113]. Epidermal growth factor receptor-tyrosine kinase inhibitors (EGFR-TKIs) are widely used in anti-tumor therapy for non-small cell lung cancer (NSCLC) [114]. HGF (hepatocyte growth factor) overexpression is a major factor contributing to acquired resistance caused by EGFR-TKI [115]. NCTD can overcome HGF-induced EGFR-TKI resistance in EGFR-mutant lung cancer cells by inhibition of the Met/PI3K/Akt pathway [116]. Therefore, NCTD may be a potential agent to reverse MDR (Table 5).

**Table 5:** Summary of related research on NCTD overcoming multidrug resistance

Cancers	Cell lines	Basic mechanisms	Pathways	Accompanying roles	Experiment	References
Oral cancer	SAS, Ca9-22	Activation of caspase-9, enhancing Bax, downregulating Bcl-2, Bcl-XL			In vitro	[108]
Breast cancer	MCF-7S, MCF-7R, MDA-MB-231, BT-474		Inhibiting Shh signaling and expression of its downstream mdr-1/P-gp expression		In vitro	[103]
	MDA-MB-231, MDA-MB-468, MDA-MB-415, AU565	Inhibiting SMAC mimetic Birinapant-mediated cell viability and promoting apoptosis and cell death; reducing c-FLIP; enhancing Birinapant-triggered caspase-8/caspase-3, Inhibiting caspase-8	Downregulation of c-FLIP	With SMAC mimetics promoting Birinapant-mediated anticancer activity		[172]
Hepatocellular cancer	Multiple HCC cell lines	Inducing transcriptional repression of Mcl-1 and enhancing ABT-737-mediated cell viability inhibition and apoptosis; activation of mitochondrial apoptosis pathway, involving cytosolic release of cytochrome *c*, cleavage of caspase-9, -3	Enhancing ABT-737-induced apoptosis by transcriptional repression of Mcl-1	Enhancing ABT-737 therapeutic efficacy	In vitro	[111]
	HepG2, SMMC-7721	ABT-737 plus NCTD have stronger proliferation inhibition, greater apoptosis induce and stronger Mcl-1 inhibiting, thus enhancing the release of cytochrome *C* and ABT-737 inducing apoptosis		With ABT-737 solving resistance of ABT-737 to liver cancer		[112]
Neuroblastoma	SH-SY5Y CHLA-119	Enhancing ABT-263-mediated apoptosis, inhibiting cell viability and clonal formation; upregulating Noxa with cytosolic release of cytochrome *c*, activation of caspase-9, -3, and cleavage of PARP	Enhancing ABT-263-mediated anticancer activity by upregulation of Noxa		In vitro	[113]
Hepatocellular cancer; Cervical cancer	HepG2 Hela	Inhibiting PTX-induced Cdc6 up-regulation, maintaining Cdk1 activity, and repressing Cohesin/Rad21 cleavage, thus reducing mitotic slippage and overcoming PTX resistance	Reducing mitotic slippage and overcoming PTX resistance via inhibiting Cdc6		In vitro	[155]
Pancreatic cancer	PANC-1, CFPAC-1	Repressing cell growth and stemness marker CD44, CD24, EPCAM, CD44(+)/CD24(+)/EPCAM(+) proportion, and β-catenin pathway-dependent manner; strengthening the cytotoxicity of gemcitabine and erlotinib	Repressing the stemness of pancreatic cancer cells through repressing β-catenin pathway, strengthening the cytotoxicity of gemcitabine, erlotinib	Strengthening the cytotoxicity of gemcitabine, erlotinib	In vitro	[173]
NSCLC	PC-9 HCC827	Reversing resistance to EGFR-TKIs induced by exogenous and endogenous HGF in EGFR mutant lung cancer cells via inhibiting the Met/PI3K/Akt pathway; NCTD plus gefitinib regressing tumor growth and Akt phosphory in vivo	Inhibition of Met/PI3k/Akt pathway	With EGFR-TKIs in vitro, with gefitinib in vivo	In vitro In vivo	[116]
Lymphoma	Multiple myeloma cells	Induction of G2/M arrest; down-regulating IKKα and p-IκBα	Inactivation of NF-kB signaling pathway	Enhancing bortezomib- antimyeloma activity	In vitro In vivo	[174]

### Promoting tumor cell demethylation

Tumorigenesis is a process of interaction between genetic and epigenetic mechanisms. DNA methylation is an important epigenetic regulator closely related to the occurrence and development of tumors [117]. Abnormal DNA methylation is involved in the pathogenesis of tumors. DNA hypomethylation promotes gene expression, while DNA hypermethylation inhibits gene expression [118, 119]. Hypermethylation of RASSF1A (a tumor suppressor gene) results in loss of function in human tumor cells [120]. It was reported that NCTD can inhibit RASSF1A methylation and inducing its re-expression in hepatocellular cancers [121]. Moreover, the Wnt/β-catenin signaling pathway is closely related to a variety of neoplastic diseases and is activated in tumor formation [122, 123]. Wnt inhibitory factor-1 (WIF-1), as a Wnt antagonist, has the function of inhibiting Wnt signal transduction. And due to hypermethylation of the promoter, WIF-1 silencing occurs in some tumor cells [124]. Studies have demonstrated that NCTD can activate WIF-1 to inhibit Wnt signaling pathway through promoter demethylation in NSCLC and glioma cells [125, 126] (Table 6).

**Table 6:** Studies of NCTD on promoting demethylation, modulating immune response and some other anticancer activities

Anticancer activities	Cancers	Cell lines	Basic mechanisms	Pathways	Accompanying roles	Experiment	Referencess
Promoting demethylation	NSCLC		Inhibiting proliferation, invasion, migration; inducing apoptosis and cell-cycle arrest; blocking β-beta-catenin; altering Bax, caspase-3, Bcl-2; activating WIF-1 and SFRP1; promoting WIF-1 demethylation, thus inhibits Wnt signal pathway	Promoting demethylation of WIF-1	Activating WIF-1 and SFRP1	In vitro	[125]
	Glioma	LN229 U251	Inhibiting proliferation, migration, invasion; inducing apoptosis and G2 phase cell-cycle arrest; downregulating Bcl-2, activating caspase-3; promoting WIF-1 and its demethylation; suppressing Wnt/β-catenin signaling, cyclin B1, and β-catenin/TCF-4; Bcl-2 and cleaved caspase-3	Inhibiting Wnt/β-catenin pathway via promoting WIF-1 demethylation	Activating WIF-1 and SFRP1	In vitro	[126]
	Hepatocellular cancer	HepG2	Inhibiting proliferation and RASSF1A methylation in a dose-dependent manner	Inhibiting RASSF1A methylation		In vitro	[121]
Modulating immune responses		Macrophages	Promoting the phosphorylation of AKT/p65 and transcriptional activity of NF-κB	Upregulation of AKT/NF-κB signaling pathway		In vitro In vivo	[127]
		Peripheral blood mononuclear cell (PBMC)	Blocking PHA-induced cyclins D3, E, A and B and IL-2 mRNAs expression; improving production of cyclin D3, E, A and B and IL-2; Cell cycle G0/G1 arrest; blocking cell proliferation			In vitro	[128]
Suppressing tumor glucose oxidative metabolism		Morris Hepatoma 7777	Suppressing tumour 14C-labelled glucose oxidative metabolism in rat Morris hepatoma			In vitro In vivo	[130]
Inhibiting NAT activity	Hepatocellular cancer	HepG2	NAT activity on acetylation of 2-aminofluorene (AF) and p-aminobenzoic acid (PABA) were examined, inhibiting NAT activity			In vitro	[131]
The effect on leukemic stem cells	Acute myeloid leukemia	MV4-11	Decreasing HLF, inducing apoptosis by modulating HLF, SLUG, NFIL3 and c-myc, thereby inducing p53 and the mitochondrial caspase cascade, producing no myelosuppression			In vitro In vivo	[4]
Modulating macrophage polarization	Hepatocellular cancer	HepG2, mouse hepatoma H22, BMDM Raw 264.7	Inhibiting tumor growth, survival and invasion, decreasing a shift from M2 to M1 polarization and CD4+/CD25+ Foxp3 T cells in HCC microenvironment; inhibiting STAT3; enhancing M1 polarization through increasing miR-214 expression; inhibited β-catenin	Through miR-214 modulating macrophage polarization		In vitro In vivo	[23]

### Modulating immune responses

The immune system plays a very important role in the development of tumors. The inflammatory response is a common and serious complication due to the continued damage to the immune system by the cancer itself and anti-cancer drugs. NCTD positively regulates macrophage-mediated immune responses via the AKT/NF-κB signaling pathway, helping to clear invading pathogens [127]; NCTD also reduces tissue inflammation by suppressing PBMC (human peripheral blood mononuclear cells) proliferation and cytokine gene expression and production [128]. In addition, the increased production of IL-10 will block the effect of specific T lymphocytes on tumor cells [129], and NCTD inhibits the production of IL-10 in PBMC induced by PHA (phytohemagglutinin) [128] (Table 6).

### Others

NCTD has also been reported to have some other anticancer activities, including inhibition of tumor glucose oxidative metabolism [130]; inhibition of NAT (*N*-acetyltransferase) activity [131]; regulation of macrophage polarization [175]; regulation of leukemia stem cell activity [4] (Table 6). Due to the lack of relevant researches, it is necessary to further verify the relevant mechanisms and applications in the clinic.

## Discussion

In recent years, the anti-tumor effect of TCMs has aroused extensive attention. However, due to the complexity of components, difficulty in extraction and high toxicity, the clinical application of many anti-tumor TCMs is limited. NCTD, as a demethylation product of CTD, can be extracted from CTD or synthesized artificially at a low cost. In addition, its physical and chemical properties are clear, so it is convenient for basic and clinical research. These prerequisites are helpful for the promotion of NCTD in clinical practice.

On the basis of summarizing the relevant literature, we found that there are two main ways of clinical application of NCTD. First of all, NCTD can be used as an anti-tumor drug alone in the treatment of liver cancer, gastric cancer and other tumors, especially for advanced malignant tumors that have lost the opportunity of operation. Secondly, it is used as an adjuvant of other anti-tumor drugs, which is currently the most important way for NCTD applied in clinic. Some studies have shown that the combination of NCTD with other anticancer drugs, or as an adjuvant to chemotherapy or interventional therapy, can help to improve the efficacy, increase the tolerance of patients, reduce side effects, and improve the prognosis [28, 30, 33].

Adverse reactions and serious complications of NCTD are rare. Gastrointestinal symptoms such as nausea and vomiting may occur when the oral dose or injection is excessive. A study has shown that patients with advanced liver cancer who take NCTD more than 45 mg/day will have significant gastrointestinal response [25]. It has also been reported that when the dosage of NCTD reaches 600 mg, the patients may have slight gastrointestinal symptoms, but it will be relieved soon after the drug is stopped or the alkaline agent is taken [27]. A large number of clinical studies have proven that patients treated with NCTD have no obvious symptoms of urinary irritation, no adverse effects on liver and renal function, and no myelosuppression [27, 28, 32].

Among the three routes of administration, oral administration and intravenous administration are simple and safe. The disadvantage is that the drug is eliminated quickly in the body, resulting in poor anti-tumor effect. It is reported that the half-life of NCTD in blood is short, only about 0.26 h [17]. Local injection is mainly used for some solid tumors, especially for advanced liver cancer which can not be treated by surgery. Compared with the former two, this method has better curative effect. However, due to the invasive operation, there are some risks such as bleeding, cancer rupture and so on.

NCTD has the disadvantages of poor water solubility, short half-life and low tumor targeting efficiency, which limits its clinical application [132, 176]. Therefore, a variety of NCTD analogues have been developed to improve the clinical applicability and efficacy. These NCTD analogues can be divided into two categories: new NCTD reagents and drug delivery systems. For example, it has been reported a new type of NCTD conjugate recently, called CNC conjugates (NCTD-conjugated carboxymethyl chitosan). Compared with the same dose of free NCTD, CNC conjugates have higher therapeutic concentration and longer half-life. It can not only enhance the inhibitory effect on cancer cells, but also reduce side effects [177, 178]. In addition, some other NCTD derivatives and liposomes, such as NOC15 (*N*-farnesyloxy-norcantharimide) [179] and SG-NCTD-LIP (NCTD-loaded liposomes modified with stearyl glycyrrhetinate) [176], also can effectively improve the anticancer activity and reduce the toxicity of NCTD. However, although these studies have shown that NCTD analogues have a very broad application prospect, most of the existing NCTD analogues have no obvious selectivity for tumors and targets. And it should be noted that most of the relevant researches are in the stage of basic research at present, whether these NCTD analogues can be applied to clinical needs to be confirmed by a large number of clinical experiments.

## Conclusions

Collectively, NCTD, as a demethylation derivative of traditional Chinese medicine, has been clinically used to treat cancer patients, and is gradually believed as a useful adjunct anticancer drug, especially for the patients with mid-advanced and postoperational recurrent cancers. The underlying molecular mechanisms of NCTD anticancer activities maybe “multi-factor”, “more targets” and “multi-points priming” mechanisms, include inhibiting proliferation, inducing apoptosis, inhibiting tumor invasion and metastasis, anti-neoangiogenesis (including anti-angiogenesis and anti-VM), anti-lymphangiogenesis, overcoming multiple drug resistance, promoting tumor cell demethylation, modulating immune responses and so on. Numerous clinical applications and drug experiments have also demonstrated that NCTD has effective and “multi-factor” anticancer activities, especially in apoptotic inducement in human cancer cells by “more targets” and “multi-points priming” mechanisms. But other mechanisms of NCTD’s anticancer effects such as anti-angiogenesis, anti-VM, anti-lymphangiogenesis as well as overcoming multiple drug resistance are seldom reported. It is necessary to improve the relevant research, which is of great significance for the development of NCTD as a potential chemotherapeutic agent.

## Data Availability

All available data and material can be accessed.

## Abbreviations

TCM: Traditional Chinese medicine
NCTD: Norcantharidin
CTD: Cantharidin
IVT: Interventional therapy
TACE: Transcatheter arterial chemoembolization
MNC: Mononuclear cells
Bcl-2: B-cell lymphoma-2
PP2A: Protein phosphatase 2A
Cdc6: Cell division cycle protein 6
PCNA: Proliferating cell nuclear antigen
MAPK: Mitogen-activated protein kinase
ERK: Extracellular regulated protein kinases
JNK: Jun N-terminal kinase
PI3K: Phosphoinositide 3 kinase
NF-κB: Nuclear factor-kappa B
MMPs: Matrix metalloproteinases
STAT1: Signal transducers and activators of transcription 1
Sp1: Specificity protein 1
EMT: Epithelial–mesenchymal transition
YAP: Yes-associated protein
VM: Vasculogenic mimicry
VEGF: Vascular endothelial growth factor
TSP: Thrombospondin
Ang: Angiogenin
TIMP: Tissue metalloproteinase inhibitor
VEGFR: Vascular endothelial growth factor receptor
Ln-5γ2: Laminin 5γ2
EphA2: Ephrin type a receptor 2
FAK: Focal adhesion kinase
MT1-MMP: Membrane type 1-matrix metalloproteinase
HLECs: Human lymphatic endothelial cells
HCACs: Human colonic adenocarcinomas
MDR: Multi-drug resistance
Shh: Sonic hedgehog
EGFR-TKIs: Epidermal growth factor receptor- tyrosine kinase inhibitors
NSCLC: Non-small cell lung cancer
HGF: Hepatocyte growth factor
WIF-1: Wnt inhibitory factor-1
PBMC: Peripheral blood mononuclear cells
PHA: Phytohemagglutinin
NAT: *N*-acetyltransferase
CNC conjugates: NCTD-conjugated carboxymethyl chitosan
NOC15: *N*-farnesyloxy-norcantharimide
SG-NCTD-LIP: NCTD-loaded liposomes modified with stearyl glycyrrhetinate
